# Severe hypercalcemia as a form of acute lymphoblastic leukemia presentation in children

**DOI:** 10.5935/0103-507X.20150067

**Published:** 2015

**Authors:** Andreia Luís Martins, Marta Moniz, Pedro Sampaio Nunes, Clara Abadesso, Helena Cristina Loureiro, Ximo Duarte, Helena Isabel Almeida

**Affiliations:** 1 Pediatric Intensive Care Unit, Hospital Prof. Doutor Fernando Fonseca, EPE - Amadora, Portugal.; 2 Department of Child and Adolescent Oncology, Instituto Português de Oncologia Lisboa, Francisco Gentil, EPE - Lisbon, Portugal.

**Keywords:** Hypercalcemia, Precursor T-cell lymphoblastic leukemia-lymphoma, Hemodiafiltration, Case reports

## Abstract

Hypercalcemia is a rare metabolic disorder in children and is potentially fatal. It has a wide differential diagnosis, including cancer. Here, we report the case of a previously healthy 3-year-old who was admitted to the emergency room with fatigue, hyporeactivity, fever and limping gait that had evolved over 5 days and that was progressively worsening. On examination the patient was unconscious (Glasgow coma score: 8). Laboratory tests indicated severe hypercalcemia (total calcium 21.39mg/dL, ionized calcium 2.93mmol/L) and microcytic anemia. Hyperhydration was initiated, and the child was transferred to the pediatric intensive care unit. Continuous venovenous hemodiafiltration with calcium-free solution was instituted, which brought progressive normalization of serum calcium and an improved state of consciousness. Zoledronate was administered, and metabolic and infectious causes and poisoning were excluded. The bone marrow smear revealed a diagnosis of acute lymphoblastic leukemia. Hypercalcemia associated with malignancy in children is rare and occurs as a form of cancer presentation or recurrence. Continuous venovenous hemodiafiltration should be considered in situations where there is imminent risk to life.

## Abstract (pt)

A hipercalcemia é um distúrbio metabólico raro em pediatria, potencialmente fatal, apresentando um vasto diagnóstico diferencial, incluindo neoplasias. Relatamos aqui o caso de uma criança de 3 anos, previamente saudável, admitida no serviço de urgência por fadiga, hiporreatividade, febre e claudicação da marcha com 5 dias de evolução, de agravamento progressivo. À observação, apresentava-se inconsciente (escore de coma Glasgow: 8). Laboratorialmente, apresentava hipercalcemia grave (cálcio total 21,39mg/dL, ionizado 2,93mmol/L) e anemia microcítica. Iniciou hiper-hidratação e foi transferido para a unidade de cuidados intensivos pediátricos. Instituiu-se hemodiafiltração venovenosa contínua com soluto livre de cálcio, ocorrendo a progressiva normalização da calcemia, com melhoria do estado de consciência. Administrou-se zolendronato. Excluíram-se causas metabólicas, infecciosas e intoxicação. O mielograma permitiu o diagnóstico de leucemia linfoblástica aguda. A hipercalcemia associada à malignidade em pediatria é rara, ocorrendo como forma de apresentação da neoplasia ou na recorrência desta. Em situações com risco de vida iminente, deve se considerar hemodiafiltração venovenosa contínua.

## INTRODUCTION

Hypercalcemia is an uncommon metabolic disorder in children. The differential diagnosis is complex and varies with age at presentation. Metabolic, nutritional, drug-induced, genetic, inflammatory and neoplastic factors may also be involved.^(1)^

Although common in adults, malignancy-associated hypercalcemia (MAH) is a rare complication at pediatric age and occurs in 0.4 to 1.3% of cancers, of which acute lymphoblastic leukemia is the most common in this age group.^(2,3)^

Treatment of MAH consists in the treatment of the underlying malignancy. In severe and persistent hypercalcemia, the initial approach is hyperhydration.^(4)^ As a part of standard treatment, prednisolone is effective in cases of moderate severity.^(4)^ Calcitonin is often reported as a treatment for pediatric MAH but has a modest hypocalcemic effect and is not marketed in Portugal. Bisphosphonates have been extensively studied and are effective in adult MAH. However, due to the rarity of the disease in children and the potential adverse effects with respect to osteogenesis, studies of efficacy and safety in this age group are limited.^(4)^ Nonetheless, small case series have confirmed its effectiveness.^(5)^ Severe symptomatic hypercalcemia requires emergency correction with continuous venovenous hemodiafiltration.

## CASE REPORT

A 3-year-old male child weighting 16kg with unremarkable past medical history presented with tiredness that had evolved over 1 week. Five days before admission, he started fever, left coxalgia and limping gait in the context of recent trauma. Due to symptom maintenance, the child was re-evaluated 3 days before admission. Imaging and laboratory studies did not suggest osteoarticular infection, and the child was given symptomatic treatment. Since the clinical picture persisted and was, accompanied by prostration, hyporeactivity and refusal to eat, he returned to the emergency room.

On admission, the patient was unconscious (Glasgow coma score: 8) with the maintenance of osteotendinous reflexes. He was hemodynamically stable and did not present any other alterations, such as rash, blood dyscrasia, lymphadenopathy, hepatomegaly or splenomegaly.

Laboratory evaluation revealed compensated metabolic alkalosis (pH of 7.41, partial pressure of carbon dioxide [PaCO_2_] of 48.7mmHg, bicarbonate [HCO_3_] of 32.5mmol/L and base excess of 9.6) and severe hypercalcemia (total calcium 21.8mg/dL, ionized calcium 2.93mmol/L). Other evaluations are shown in table 1. Craniocephalic computed tomography and renal, abdominal and hip joint ultrasound showed no significant changes.

**Table 1:** Evaluation performed on admission

Analysis	Result	Reference value
Hemoglobin (g/dL)	9.3	11.5 - 11.5
Hematocrit (%)	26.6	34 - 43
Mean corpuscular volume (fL)	73.1	75 - 90
Leukocytes (/uL)	5,500	4,000 - 12,000
Neutrophils (/uL)	2,300	
Lymphocytes (/uL)	2,500	
Platelets (/uL)	186,000	150,000 - 350,000
C-reactive protein (mg/dL)	7.96	< 0,3
Urea (mg/dL)	60	17 - 38.5
Creatinine (mg/dL)	0.83	0.5 - 1.1
Albumin (g/dL)	3.3	3.6 - 5.2
Aspartate aminotransferase (UI/L)	42	10 - 47
Alanine aminotransferase (UI/L)	48	24 - 49
Lactate dehydrogenase (UI/L)	739	155 - 280
Alkaline phosphate (UI/L)	121	191 - 450
Serum inorganic phosphorus (mg/dL)	3.1	4.0 - 6.0
Serum magnesium (mg/dL)	1.1	1.7 - 2.4
Serum potassium (mmol/L)	2.52	3.5 - 5.0
Serum sodium (mmol/L)	133	135 - 145

**Figure 1:**
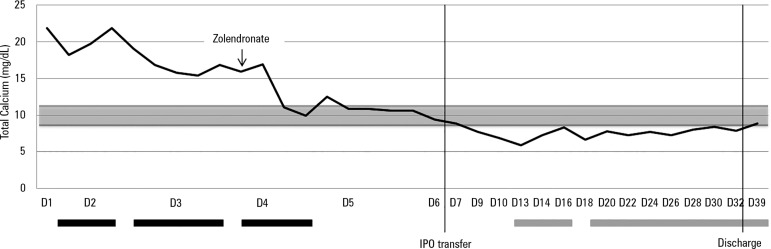
Evolution of total calcemia during hospitalization in the pediatric intensive care unit. Grey area: total calcium reference value limits; black bars: period under continuous venovenous hemodiafiltration; grey bars: period under calcium supplementation. IPO - *Instituto Português de Oncologia*.

Given the clinical and laboratory severity of hypercalcemia, on suspicion of osteoarticular infection, intravenous hyperhydration was initiated (2,500mL/m^2^/day), and antibiotics were given (flucloxacillin and gentamicin). The child was transferred to the pediatric intensive care unit. Continuous venovenous hemodiafiltration was initiated after a 6.5F hemodialysis central venous catheter was placed in the right femoral vein. An HF20 filter was used and priming was performed with 5,000 UI of heparin in 1L of 0.9% sodium chloride. Continuous venovenous hemodiafiltration was programmed in accordance with the pediatric protocol (25 - 40mL/kg/h = 1/3 dialysis fluid + 2/3 fluid replacement (2/3 prefilter + 1/3 post-filter)). Ultrafiltrate was calculated according to the desired fluid balance.^(6)^ A replacement and calcium-free dialysis solution was used (Prism0Cal^®^, Gambro - Lund, Sweden). Regional anticoagulation was performed with machine-perfused unfractionated heparin, the dose of which was adjusted according to the patient's activated partial thromboplastin time. The technique was maintained for 72 hours and took place without complications. As a therapeutic supplement, intravenous zoledronate (0.025mg/kg) was administered on the third day of hospitalization. There was a progressive decrease in total and ionized calcium levels and an improved state of consciousness (Figure 1).

The investigation revealed low (unmeasurable) serum intact parathyroid hormone (PTH) and excluded metabolic and infectious causes and vitamin or drug poisoning (Table 2). Skeletal radiography excluded osteolytic lesions.

**Table 2:** Laboratory tests performed

Analysis	Result	Reference value
PTH intact	< 20	
PTHrp (pmol/L)	1.2	< 2.0
1.25 (OH)_2_D (pmol/L)	2	39 - 193
25 (OH)D (ng/dL)	12.7	30 - 100
Retinol (ng/dL)	21	30 - 70
ACE (U/L)	20.10	12 - 68
EBV-VCA IgM/IgG	Negative/positive	
Parvovirus B19 IgM/IgG	Negative/positive	
CMV IgM/IgG	Negative/positive	
*Mycoplasma pneumoniae* IgM/IgG	Negative/negative	
HIV 1/2	Negative	
FT3 (pg/mL)	2.31	4.0 - 7.10
FT4 (ng/dL)	0.56	0.9 -1.70
TSH (mUI/L)	0.496	0.8 - 7.5
Total proteins (g/dL)	5.6	6 - 8
Albumin (g/dL)	2.53	3.75 - 5.01
Uric acid (mg/dL)	9.4	3.0 - 5.5

PTH - parathyroid hormone; PTHrp - parathyroid hormone-related protein; 1.25 (OH)_2_D - 1.25-dihydroxy-vitamin D; 25(OH)D - Vitamin D in the form of 25-hydroxyvitamin D; ACE - angiotensin converting enzyme; EBV-VCA - viral capsid antigen of the Epstein-Barr virus; CMV - cytomegalovirus; HIV - human immunodeficiency virus; FT3 - free T3 hormone; FT4 - free T4 hormone; TSH - thyroid stimulating hormone.

During hospitalization, progressive pancytopenia (hemoglobin 7.7g/dL, leukocytes 2200/uL, platelets 67000/uL) was identified and bone marrow examination was performed, which confirmed precursor B-cell acute lymphoblastic leukemia.

After diagnosis, the child was transferred to a pediatric oncology referral center, where, after further review, remission induction therapy was started (2005 ALL-DFCI protocol of the Dana-Farber Cancer Institute). The complementary evaluation by flow cytometry revealed 69% infiltration of B lymphoblasts. During hospitalization, there were periods of asymptomatic hypocalcemia (minimum 5.9mg/dL at day 10 post-zoledronate), which required intravenous correction. A bone marrow smear performed 4 weeks after the beginning of induction therapy confirmed complete remission both morphologically and by molecular biology.

Currently, approximately 10 months after starting treatment, the child remains in remission.

## DISCUSSION

Hypercalcemia is a potentially fatal disorder, regarding its neurological and cardiac complications. The treatment includes hyperhydration, bisphosphonates and treatment of the underlying disease.^(1)^ Occasionally, rapid correction of the disturbance becomes crucial, particularly in the setting of loss of consciousness or when the hypercalcemia is refractory to conventional measures. In such situations, the use of continuous venovenous hemodiafiltration has been identified as an effective treatment.^(7,8)^ Its successful use in severe hypercalcemia has been reported in adults,^(8-11)^ but the use of the technique in pediatrics has rarely been described in the literature.^(7,12)^ In this case report, due to severe hypercalcemia on admission, the use of dialysis solution with calcium-free replacement was chosen. Regular analytical calcemia controls were performed in order to avoid a sudden decrease and below-normal values. The renal replacement therapy settings were set in order to provide a gradual decrease in serum calcium, thereby avoiding complications such as circuit clotting. As the patient did not present spontaneous diuresis, it was decided to program losses to ensure a neutral fluid balance. Following clinical and laboratory stabilization, zoledronate was introduced to maintain normocalcemia, as the effect of continuous venovenous hemodiafiltration is temporary.^(11)^ Continuous monitoring of serum calcium levels was assured due to the risk of hypocalcemia observed in this case.^(13)^

The etiological investigation suggested an independent PTH mechanism. Metabolic and infectious causes and vitamin or drug poisoning were excluded. Progressive pancytopenia led to the suspicion of MAH, which ultimately led to the final diagnosis.

The pathogenesis of MAH includes the stimulation of bone resorption, mediated by proteins and cytokines produced by the tumor cells or by the tumoral microenvironment. Two distinct mechanisms are described, which include hypercalcemia by local osteolytic lesions (bone metastasis) and humoral hypercalcemia by the activation of RANK-RANKL (receptor activator of nuclear factor κB and its ligand). Parathyroid hormone-related protein (PTHrP) is the most frequently involved mediator, but other mediators, such as interleukin (IL)-1, IL-6, tumor necrosis factor alpha (TNF-α), transforming growth factor beta (TGF-β), prostaglandins and even calcitriol and ectopic PTH production may be involved.^(4)^

In acute lymphoblastic leukemia, an association with hypercalcemia in patients with t (17;19) has been reported, suggesting the possible induction of PTHrP.^(3,14)^ In this case, this cytogenetic abnormality was not observed, and high levels of PTHrP were not detected, thus excluding this mechanism as the *primum movens* of hypercalcemia.

## CONCLUSION

The described case shows an infrequent complication, not only at pediatric age, but also in children with oncological diseases, suggesting that this metabolic emergency unveils of the underlying disease. Continuous venovenous hemodiafiltration with calcium-free solution as a first-line treatment in cases of severe and symptomatic hypercalcemia was found to be effective in the rapid induction of normocalcemia and neurological improvement, buying valuable time until maintenance treatment focused on the etiology can exert a sustained effect.
